# The Effect of Single-Dose Ougan Juice Application on the Pharmacokinetics of Erlotinib

**DOI:** 10.1155/2021/6679082

**Published:** 2021-06-11

**Authors:** Jin-zhao Yang, Yi Song, Jian-hua Xiong, Yu-xian Lin, Congcong Wen, Yu Li, Yunfang Zhou

**Affiliations:** ^1^Department of Pharmacy, The Third Affiliated Hospital of Shanghai University (Wenzhou People's Hospital), Wenzhou 325000, China; ^2^School of Pharmaceutical Sciences, Wenzhou Medical University, Wenzhou 325035, China; ^3^Laboratory Animal Centre, Wenzhou Medical University, Wenzhou, China; ^4^The Laboratory of Clinical Pharmacy, The People's Hospital of Lishui, Lishui 323000, China

## Abstract

The aim of our study was to investigate the effects of single-dose Ougan (Citrus reticulata cv. Suavissima) juice application on the pharmacokinetics of erlotinib in vivo. Twelve Sprague-Dawley rats were randomly divided into the Ougan juice and control groups (*n* = 6 each). The rats were given a single dose of 1 mL/100 g Ougan juice or 1 mL/100 g normal saline (NS) by intragastric administration, followed by a single oral administration of 20 mg/kg erlotinib. The plasma concentration of erlotinib in rats was determined using ultra performance liquid chromatography–tandem mass spectrometry (UPLC-MS/MS). Erlotinib-d6 was used as the internal standard for chromatographic analysis on the UPLC BEH C18 analysis column (2.1 mm × 50 mm, 1.7 *μ*m). The mobile phase was composed of acetonitrile and 0.1% formic acid eluting by gradient. Different pharmacokinetic (PK) parameters of erlotinib were calculated. The Ougan juice promoted the absorption of erlotinib and reduced the clearance of the drug. The area under the curve of erlotinib in the single-dose Ougan juice pretreatment group was approximately 1.87 times higher, and the maximum blood concentration (Cmax) was approximately 1.34 times higher than that in the control group. The mean residence time of erlotinib in the Ougan juice group was larger, and the clearance rate was smaller than those in the control group; the difference was statistically significant (*P* < 0.05). Ougan juice affected the PK spectrum of erlotinib in rats by improving the bioavailability of the drug and significantly increasing its plasma concentration.

## 1. Introduction

In recent years, the interaction between food and drugs has caused widespread concern [1]. Food or a substance in it may alter the pharmacokinetics (PK) or pharmacodynamics of a drug, especially an oral drug [2, 3]. Because the bioavailability of most drugs is related to clinical efficacy, it is an important PK parameter [4]. Ougan (*Citrus reticulata* cv. *Suavissima*) is the main local citrus variety in Zhejiang Province, China, and has a pleasant taste and high nutritional value. It is one of the first choices of complementary fruits. According to research [5], Ougan not only has antihypertensive, hypothermia, coronary artery expansion, and hypoxia resistance [6] but also has potential antitumor effects [7]. Ougan juice contains various bioactive substances, such as vitamin B, vitamin C, and peroxidase [8], which may interact with many oral drugs and change their PK. Compared with most citrus, antioxidants such as flavonoids are the main nutritional value of Ougan juice. One study has indicated that a single dose of Ougan juice has no effect on the activity of cytochrome (CYP)450, an enzyme essential for the production of cholesterol, steroids, prostacyclins, and thromboxane A2 and for the detoxification of foreign chemicals and the metabolism of drugs in rats, while multiple doses of Ougan juice could induce CYP2C9 and inhibit the activity of CYP1A2 and CYP2C19, but has no effect on CYP2D6 [9]; however, studies on the effect of Ougan juice on CYP3A4 enzyme activity is lacking.

Non small–cell lung cancer (NSCLC) is one of the most common of all cancers, with high morbidity and mortality [10]. Erlotinib, a tyrosine kinase inhibitor (TKI) [11], is commonly used as an oral targeted therapy to treat the epidermal growth factor receptor gene- (*EGFR-*) mutant NSCLC [12, 13]. It is ~60% bioavailable, and the peak concentration is reached in 4 h. Food can significantly increase the bioavailability to ~100% [14, 15]. In vivo, erlotinib is mainly metabolized in the liver by CYP3A4, and a small amount is metabolized by the CYP1A2, -2C8, and -2D6 pathways [16, 17]. Any drugs metabolized by these enzymes, as well as enzyme inducers or inhibitors, may interact with erlotinib and have certain effects on erlotinib clearance [15, 18]. As a nutritional fruit often consumed by cancer patients, Ougan's interaction with erlotinib remains unclear; however, studying the PK of this fruit's juice on erlotinib is of great significance for helping identify the protocol for rational use of this drug.

## 2. Materials and Methods

### 2.1. Chemicals and Reagents

Ougan juice was supplied by the Department of Pharmacy, Wenzhou People's Hospital (Wenzhou, China). Ougan juice contains a large number of flavonoids, including 4 kinds of flavanone glycosides (naringin, hesperidin, neohesperidin, and citrinin) and 4 kinds of polymethoxy flavonoids (sweet orange flavone, tangeretin, tangeretin, and 5-nor tangeretin); Ougan also contains phenolic substances and carotenoids and other antioxidant active functional components.

Erlotinib (purity >98%) and erlotinib-d6 used as the internal standard (IS; purity >98%) were supplied by the Chinese Biopharmaceutical Institute (Beijing, China). Methanol and acetonitrile (high performance liquid chromatography [HPLC] grade) were obtained from the Merck Company (Darmstadt, Germany). Ultrapure water was obtained using a Milli-Q purification system (Millipore, Bedford, MA, USA). Sprague-Dawley rats (200–220 g each) were acquired from the Laboratory Animal Centre of Wenzhou Medical University, China, and were placed in the laboratory facility for 2 weeks to adapt to the environment before beginning the experiment.

### 2.2. Instrumentation and Conditions

A UPLC-tandem mass spectrometer (-MS/MS) system using the ACQUITY H-Class UPLC and XEVO TQS-micro Triple Quadrupole Mass Spectrometer (Waters Corporation, Milford, MA, USA) was used. Masslynx 4.1 (Waters Corporation) was used for data acquisition and instrument control.

The UPLC-MS/MS column was UPLC BEH C18 (2.1 mm × 50 mm, 1.7 *μ*m), and the column temperature was set at 40°C. The mobile phase was composed of acetonitrile and 0.1% formic acid, with gradient elution at a flow rate of 0.6 mL/min. The gradient elution was as follows: 0–0.2 acetonitrile 10%, 0.2–1.5 min, linear acetonitrile from 10 to 80%; 1.5–2.0 min, acetonitrile 80%; 2.0–2.1 min, linear acetonitrile from 80 to 10%; and 2.1–3.0 min, acetonitrile 10% [19, 20]. The gradient elution can avoid the problem of isocratic elution, the peak shape is changed, and there is little tailing.

Nitrogen was used as the desolvation gas (900 L/h) and 50 L/h cone gas. The capillary voltage was set to 1.5 kV, the ion source temperature was 150°C, and the desolvation temperature was 450°C. The electrospray ionization source (ESI), positive ion detection, quantitative analysis using multiple reaction monitoring, 394.2⟶278.1 m/z erlotinib (cone voltage 35 V, collision voltage 30 V), and internal standard 400.2⟶278.1 m/z (cone voltage 35 V, collision voltage 30 V) were used in the UPLC-MS/MS system [21].

### 2.3. Preparation of Reference Solution

Erlotinib (1.0 mg/mL) and erlotinib-d6 (1.0 mg/mL) stock solutions were prepared in acetonitrile. The erlotinib stock solutions were diluted with acetonitrile to prepare a series of standard working solutions. Erlotinib-d6 stock solution was diluted with acetonitrile to prepare an acetonitrile solution containing IS erlotinib-d6 at a concentration of 50 ng/mL. All solutions were stored at 4°C.

### 2.4. Standard Curve

A sensitive and rapid UPLC-MS/MS method was established to determine the concentration of erlotinib and erlotinib-d6 in rat plasma [21, 22]. Using the peak area ratio of erlotinib to IS as the ordinate (*Y*) and the concentration of erlotinib in the plasma as the abscissa (*X*), a standard curve was drawn with a range of 1–2000 ng/mL.

### 2.5. Plasma Sample Treatment

Several 100-*μ*L plasma samples were combined with 200 *μ*L acetonitrile containing 50 ng/mL IS, vortexed for 1.0 min, and centrifuged at 13000 rpm for 10 min at 4°C, after which 100 *μ*L supernatant was collected into the inner lining pipe of a sample vial, and 2 *μ*L was injected into the UPLC-MS/MS system for analyses.

### 2.6. Method Validation

Validation methods were established in accordance with FDA guidelines for validation of bioanalytical methods. Validation items include selectivity, matrix effects, linearity, precision, accuracy, recovery, and stability.

### 2.7. Pharmacokinetic Study

Fresh Ougans were picked, peeled, squeezed and packed, and stored at -20°C. The fruit was thawed at room temperature to normal temperature before each use. The 12 SD rats were randomly divided into 2 groups (*n* = 6 per group), one being the Ougan juice group and the other being the control group. A single dose of 1 mL/100 g Ougan juice (before thawing at -20°C) and 1 mL/100 g NS was intragastric administered, respectively, to the Ougan and control groups, and both groups were intragastric administered an erlotinib solution at 20 mg/kg of body weight. Before administration (0 h) and at different time intervals after administration (i.e., 0.085, 0.17, 0.33, 0.5, 1, 2, 3, 4, 6, 8, 12, and 24 h), 0.3 mL blood was collected from the tail vein of each rat into 1.5 mL heparinized Eppendorf (EP) tubes and centrifuged at 13000 rpm for 10 min. The upper plasma was transferred into a new EP tube and frozen and stored at -20°C. The samples were used for UPLC-MS/MS analyses.

### 2.8. Statistical Analyses

Plasma concentration versus time data for each rat was analyzed by DAS( version 3.0) to obtain pharmacokinetic parameters. The PK calculation parameters were as follows: Cmax, peak time (Tmax), half-life (t1/2z), area under the curve (AUC), clearance rate (CLz/F), and apparent distribution volume (Vz/F). All data were analyzed using GraphPad Prism (GraphPad Software, Inc., La Jolla, CA, USA) and represented by the mean ± standard deviation. Statistical differences between groups were calculated using the *t-*test. For all tests, *P* < 0.05, *P* < 0.01, and *P* < 0.001 indicate significant difference between the control and experimental group.

## 3. Results

### 3.1. Method Validation

Typical UPLC-MS/MS chromatograms of blank plasma spiked with erlotinib and IS with reasonable peak time of 1.29 min and sharp peak shape. No interference of visible impurity and endogenous substances was observed.

Taking the ratio of erlotinib to the peak area of IS as the vertical coordinate (*Y*) and the plasma concentration of erlotinib as the horizontal coordinate (*X*), the linear regression equation was *Y* = 0.0018*x* − 0.0007, with a high correlation coefficient *R* =0.9983 (*n* = 6). The method produced a good linear relationship within the range of 1 ~ 2000 ng/mL. The LLOQ of erlotinib in rat plasma was 1 ng/mL.

The intraday and interday accuracy of erlotinib was 97% and 107%. The precision was less than or equal to 14%. The matrix effect ranged from 99% to 108%. The recoveries were above 93%.

The stability of erlotinib in variation condition (3 freezing and thawing cycles, room temperature for 2 h, -20°C for 30 days) was acceptable, the accuracy was within 86% and 110%, and precision was less than 15%.

### 3.2. Pharmacokinetic Study

**Figure 1:**
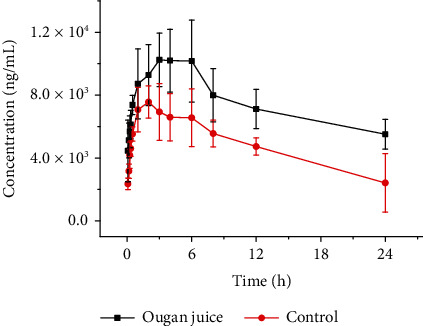
Mean plasma concentration-time curves of erlotinib in rat plasma.

Changes in the mean plasma concentration of erlotinib in the rats over time 1 d after a single dose of Ougan juice and erlotinib combined are provided in Figure 1.  Table 1 summarizes the average noncompartmental PK parameters of erlotinib. AUC and the blood concentration of erlotinib in the single-dose Ougan juice pretreatment group were significantly larger than that in the control group (Figure 1). After additional calculations using DAS 2.0, as shown in Table 1, the results showed no significant changes in t1/2, Tmax, and Vz/F of erlotinib in the single-dose Ougan juice pretreatment group compared with those in the control group (*P* > 0.05); however, AUC of erlotinib in the single-dose Ougan juice pretreatment group was approximately 1.87 times higher, and Cmax was approximately 1.34 times higher than those in the control group, and the mean residence time MRT(0-t) of erlotinib in the Ougan juice group was larger and CLz/F smaller than those in the control group; the difference was statistically significant (*P* < 0.05). The results indicated that a single dose of Ougan juice could significantly increase the absorption rate and capacity, reduce the clearance rate, and prolong the residence time of erlotinib in the body.

## 4. Discussion

TKIs can interact with a variety of clinically relevant drugs and foods [23], mostly because of changes in gastric pH and bioavailability caused by the metabolism of CYP450 isoenzymes [24]. Erlotinib is an oral reversible TKI of EGFR effective in NSCLC. Current studies have confirmed that the bioavailability of erlotinib depends on stomach acidity. Because erlotinib is weakly basic, it can exist in ionized or nonionized form. As the pH in the stomach decreases, the equilibrium shifts to a soluble ionized form of erlotinib, and drug absorption increases [25]. Ougan juice is acidic, which can directly reduce gastric pH and stimulate gastric acid secretion to promote the absorption of erlotinib, thereby significantly increasing Cmax [26]. Erlotinib is mainly metabolized by CYP3A4 enzyme in the liver and secreted by the biliary tract.  Table 1 shows that a single dose of Ougan juice can reduce the clearance rate of erlotinib, which suggests that this fruit's juice may inhibit the metabolism of erlotinib by inhibiting the activity of CYP3A4. The specific mechanism of this is unclear and needs further study.

## 5. Conclusion

We determined the effect of Ougan juice on PK of erlotinib using the UPLC-MS/MS method. The results demonstrated that a single dose of Ougan juice can significantly promote the absorption of erlotinib and may reduce the drug clearance rate by inhibiting the CYP3A4 pathway, so as to increase the bioavailability of erlotinib and significantly increase its concentration in plasma. This finding has important clinical significance of the effects of eating Ougans for cancer patients being treated with erlotinib.

## Figures and Tables

**Table 1:** The main pharmacokinetic parameters of erlotinib in two groups (*n* = 6).

Parameters	Unit	Ougan juice	Control
AUC (0-t)	Ng/mL × h	180147.19 ± 25024.12^∗∗^	107445.29 ± 31568.29
AUC (0-∞)	Ng/mL × h	390920.55 ± 77376.29^∗∗^	209227.68 ± 99667.80
MRT (0-t)	h	10.61 ± 0.60^∗^	8.56 ± 2.70
MRT (0-∞)	h	40.47 ± 15.62	29.73 ± 23.44
t1/2z	h	28.34 ± 11.10	20.28 ± 17.01
Tmax	h	3.67 ± 1.37	3.00 ± 2.12
Vz/F	L/kg	2.05 ± 0.46	2.40 ± 1.22
CLz/F	L/h/kg	0.05 ± 0.01^∗^	0.12 ± 0.05
Cmax	Ng/mL	11969.86 ± 2008.84^∗∗^	8897.41 ± 564.90

Notes: compared to the control group, ^∗∗∗^*P* < 0.001, ^∗∗^*P* < 0.01, ^∗^*P* < 0.05.

## Data Availability

The data used to support the findings of this study are included within the article.
